# Antimicrobial Peptide Trichokonin VI-Induced Alterations in the Morphological and Nanomechanical Properties of *Bacillus subtilis*

**DOI:** 10.1371/journal.pone.0045818

**Published:** 2012-09-25

**Authors:** Hai-Nan Su, Zhi-Hua Chen, Xiao-Yan Song, Xiu-Lan Chen, Mei Shi, Bai-Cheng Zhou, Xian Zhao, Yu-Zhong Zhang

**Affiliations:** The State Key Laboratory of Crystal Materials and the State Key Laboratory of Microbial Technology, Marine Biotechnology Research Center, Shandong University, Jinan, People’s Republic of China; Dalhousie University, Canada

## Abstract

Antimicrobial peptides are promising alternative antimicrobial agents compared to conventional antibiotics. Understanding the mode of action is important for their further application. We examined the interaction between trichokonin VI, a peptaibol isolated from *Trichoderma pseudokoningii*, and *Bacillus subtilis,* a representative Gram-positive bacterium. Trichokonin VI was effective against *B. subtilis* with a minimal inhibitory concentration of 25 µM. Trichokonin VI exhibited a concentration- and time-dependent effect against *B. subtilis*, which was studied using atomic force microscopy. The cell wall of *B. subtilis* collapsed and the roughness increased upon treatment with trichokonin VI. Nanoindentation experiments revealed a progressive decrease in the stiffness of the cells. Furthermore, the membrane permeabilization effect of trichokonin VI on *B. subtilis* was monitored, and the results suggest that the leakage of intracellular materials is a possible mechanism of action for trichokonin VI, which led to alterations in the morphological and nanomechanical properties of *B. subtilis*.

## Introduction

Bacterial pathogens that are resistant to conventional antibiotics are becoming more difficult to treat. Natural products are a valuable and fundamental source for new drug discovery. Among the potential candidates, antimicrobial peptides (AMPs) have recently drawn increasing interest and have been proposed to be a promising alternative to conventional antibiotics [1], [2]. AMPs exist in various species, including animals, plants, fungi and bacteria, and act in the innate defense of the organisms [3]. Investigating the antimicrobial mechanisms of the AMPs is important for their application.

Among the various AMPs, the fungi-derived cationic peptaibols are an important group that constitute a large family of approximately 850 members and could be a potential source for new antimicrobial drugs [4]. Peptaibols are characterized as linear peptides of 5–20 residues that contain a C-terminal amino alcohol, an acylated N-terminus, and a high proportion of non-standard amino acid residues, including α-amino isobutyric acid (Aib), isovaleric acid (Iva) and imino acid hydroxyproline (Hyp).

Peptaibols have been isolated from at least 23 fungal genera, and the genus *Trichoderma* and related genera are the most abundant sources of peptaibols [5], [6]. While most conventional antibiotics do not act against fungi, and fungi-killing drugs are not active against bacteria [7], peptaibols and other AMPs have excellent activity against both bacterial and fungal pathogens [7]. Moreover, earlier research has shown that peptaibols can also function as suppressors of tumor cells by inducing apoptosis and autophagy in hepatocellular carcinoma cells while causing no obvious harm to normal liver cells [8].

Alterations in the morphological and nanomechanical properties of bacteria induced by AMPs are directly related to the mechanisms of antimicrobial action of the peptaibol agents. Therefore, atomic force microscopy (AFM), which is advantageous for investigating the ultrastructural and nanomechanical properties of bacteria [9], [10], [11], is useful in researching the mechanisms of AMPs against microorganisms. However, currently published AFM studies on the antimicrobial effects of AMPs have mostly focused on Gram-negative bacteria, such as *E. coli* and *Pseudomonas aeruginosa* [12], [13], [14], [15], [16], [17], [18], [19], [20], and studies using AFM to observe the morphological and nanomechanical properties of Gram-positive bacteria treated with AMPs are rare.

Here, we report the antimicrobial effects of trichokonin VI, a peptaibol produced by *Trichoderma pseudokoningii,* on the Gram-positive bacterium *B. subtilis*. Changes to the morphological and nanomechanical properties were monitored and probed by AFM in combination with other experimental methods. The mechanism of the action of trichokonin VI on bacterial membranes is also discussed.

## Materials and Methods

### Preparation of Trichokonin VI

Trichokonin VI was prepared from *T. pseudokoningii* SMF2 using solid-state fermentation following previously described methods [21]. The purity of the prepared Trichokonin VI was confirmed by HPLC (data not shown). The purified trichokonin VI (10 mg) was first dissolved in methanol (0.1 ml) and diluted with Milli-Q water (4.9 ml) to a final concentration of 2 mg/ml as a stock solution. The stock solution of trichokonin VI was stored at 4°C.

### Preparation of Bacterial Cells

*B. subtilis* from a single colony was grown at 37°C in Mueller Hinton Broth (MHB; Hangzhou Microbial Reagent Co., Ltd.; China) overnight to a final concentration of 10^8^∼10^9^ CFU/ml. The bacterial culture was diluted with fresh MHB to a concentration of 10^6^ CFU/ml for the susceptibility test and time-killing test.

### Susceptibility Test

The minimal inhibitory concentration (MIC) was determined for trichokonin VI according to a modified microtiter-broth dilution method [22]. Trichokonin VI stock solution was diluted with Milli-Q water to a concentration of 200, 100, 50, 25, 12.5 6.25, 3.12, 1.56, 0.78 and 0.39 µM. Each dilution (100 µl) was transferred to a microtiter plate well, and 100 µl of bacterial suspension (at a concentration of 10^6^ CFU/ml) was added to each cell. The final concentration of trichokonin VI was 100, 50, 25, 12.5 6.25, 3.12, 1.56, 0.78, 0.39 and 0.2 µM per well. The well that contained only growth medium was set as sterility control. The well that contained only bacterial suspension was set as growth control. Three rows were used for replicates on each microtiter plate for every concentration, and three microtiter plates were used for parallel experiment. The plates were covered with a plastic lid to avoid contamination and incubated at 37°C for 16–20 h without shaking. The MIC was defined as the lowest concentration that inhibited the visible growth of bacteria compared with the control sample. The experiment was repeated three times.

### Time-kill Curves

The stock solution of trichokonin VI was diluted with Milli-Q water to a concentration of 1, 2 and 4 × MIC (corresponding to 25 µM, 50 µM and 100 µM), and 1 ml of the trichokonin VI solution was mixed with 1 ml of bacterial suspension in a test tube, which resulted in final trichokonin VI concentrations of 0.5, 1 and 2 × MIC (corresponding to 12.5 µM, 25 µM and 50 µM). The mixture of 1 ml bacterial suspension and 1 ml Milli-Q water was used as a control. The bacteria were incubated at 37°C with agitation. Samples were collected at predetermined time points (0, 2, 4, 6, 8, 10, 12 and 20 h). The samples were serially diluted with Milli-Q water and spread on Luria-Bertani (LB) broth agar plates, with three replicates used for every dilution of each time point. The number of viable colonies was counted after the plates were incubated at 37°C for 18–24 h.

### AFM Imaging

The *B. subtilis* cells were incubated at 37°C with trichokonin VI at 0.5, 1 and 2 × MIC (corresponding to 12.5 µM, 25 µM and 50 µM). Control samples were not treated with trichokonin VI. Sample preparation process was the same with that described in *Time-kill curves* section. Samples were collected at 0.5, 1, 2, and 5 h time points and were centrifuged at 7,000 g for 10 min. The *B. subtilis* cells were suspended in Milli-Q water. A drop (2.5 µl) of *B. subtilis* was spread onto freshly cleaved mica and air-dried at room temperature before imaging. AFM images were obtained using a Multimode Nanoscope V (Bruker AXS; German) in tapping mode, and a probe (NSC11, MikroMasch) with a cantilever length of 90 µm was used.

### Surface Roughness Analysis

The AFM data of the *B. subtilis* cells treated with trichokonin VI at the MIC (25 µM) for different time periods were flattened and used to calculate surface roughness. The surface roughness of a selected area was calculated using the NanoScope Analysis AFM software. Root mean square average roughness (*Rq*) was calculated with Equations 1.
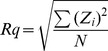
(1)
*Z_i_* is the height value at the *i*th point, and *N* is the number of points within the selected area. For each sample, roughness was measured near the center area on a bacterial cell with fixed sizes of 400 × 400 nm^2^. At least 5 different areas from different bacterial cells were measured.

### Force-curve Measurements

*B. subtilis* cells treated with trichokonin VI at the MIC for different time periods were selected for the force-curve measurements. The spring constant *k_c_* of the probe cantilever (NSC11, MikroMasch) with a length of 200 µm was determined in air using the thermal noise method and Nanoscope software. Samples were first imaged in tapping mode to identify bacterial cells. At least 10 force curves per sample were collected in contact mode near the center of the selected bacterial cell. The spring constant of the cantilever used in the force experiments was approximately 2.0 N/m, which is smaller than that used to measure the mechanical properties of bacteria in air in some other works [23], [24]. The spring constant of the cantilever was larger than the spring constant used in liquid [25], [26], [27], due to the higher rigidity of the cells in air.

### Mechanical Property Analysis

From the approaching branch of the force-distance curves, the stiffness of the bacteria could be determined. When force was applied on a softer sample, the slope of the linear portion of the approaching branch in the contact region was lower. The bacteria and cantilever could be modeled as two connected springs, and the spring constant of the bacteria could be determined by the slope (*s*) of the linear portion of the force curve [28]. The spring constant of the bacteria can be calculated according to Equation 2.
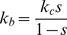
(2)


In Equation 3, *k_b_* is the spring constant of the bacteria, and *k_c_* is the spring constant of the cantilever.

The jump-off contact point of the retract branch from the force-distance curve reflects the tip-sample adhesion interaction. This adhesive force may be due to biomacromolecules and thin water films on the cellular surface. The adhesion force between the probe tip and bacterial surface could be calculated according to Equation 3.
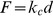
(3)


In Equation 4, *F* is the adhesion force and *d* is the deflection of the cantilever that resulted from the adhesive interaction between the tip and bacterial surface.

As the area in the retract branch from the force-distance curve below the zero force line represents the work performed by the adhesion force, the adhesion energy, which is also an indicator of bacterial surface properties, was subsequently calculated.

### Leakage of Cellular UV-absorbing Materials

To analyze the damage of the bacterial cytoplasmic membrane caused by trichokonin VI, leakage of UV-absorbing cellular substances following sample treatment was monitored. Bacterial cell suspensions were mixed with trichokonin VI to a final concentration of 0.5, 1, 2 and 4 × MIC (corresponding to 12.5 µM, 25 µM, 50 µM and 100 µM), and the controls were cells without trichokonin VI treatment. Samples were collected at different time points. The samples were centrifuged at 7,000 g for 10 min to remove the bacterial cells. The supernatants were diluted, and their absorbance at 210 nm, 260 nm and 280 nm was recorded. The absorbance of trichokonin VI at concentrations of 0.5, 1, 2 and 4 × MIC (corresponding to 12.5 µM, 25 µM, 50 µM and 100 µM) were measured to exclude its absorption. Absorbance was measured at room temperature using a UV/VIS-550 spectrophotometer (Jasco; Japan).

### Membrane Permeabilization Test

Trichokonin VI was added to *B. subtilis* cultures to a final concentration of 0.5, 1 and 2 × MIC (corresponding to 12.5 µM, 25 µM and 50 µM). Bacterial samples without treatment were used as a control. After treatment for 60 min, samples were centrifuged at 7,000 g for 10 min and washed twice in phosphate buffer. The bacterial cells were suspended in phosphate buffer to a concentration of 10^5^–10^6^ CFU/ml and incubated with SYTOX Green (to a final concentration of 2 µM) for 10 min. Fluorescence of SYTOX Green was examined by flow cytometry (FACSCalibur, Becton-Dickinson).

## Results

### Antimicrobial Susceptibility Tests

The antimicrobial activity of trichokonin VI against *B. subtilis* was determined using the broth-dilution method. Gram-positive *B. subtilis* showed susceptibility to trichokonin VI in the test. In the wells on the microtiter plates with the concentration of trichokonin VI at 12.5 µM or lower, the deposits or turbidity which indicated the growth of bacteria was visible. However, in the wells with the concentration of trichokonin VI at 25 µM or higher, the liquids in the wells were clean, and neither turbidity nor deposits could be visualized. By carefully examining all parallel experiments for the antimicrobial susceptibility tests, the MIC value of trichokonin VI to *B. subtilis* was determined to be 25±0 µM, which is smaller than those of the AMPs such as LEAP-2 [29] and Cn-AMP1 [30], similar to that of Magainin2 [29] and higher than those of LL-37 [29] and MSI-594 [31]. The error in the MIC could not be determined as it was less than the dilution factor used.

### Time-kill Curves

**Figure 1:**
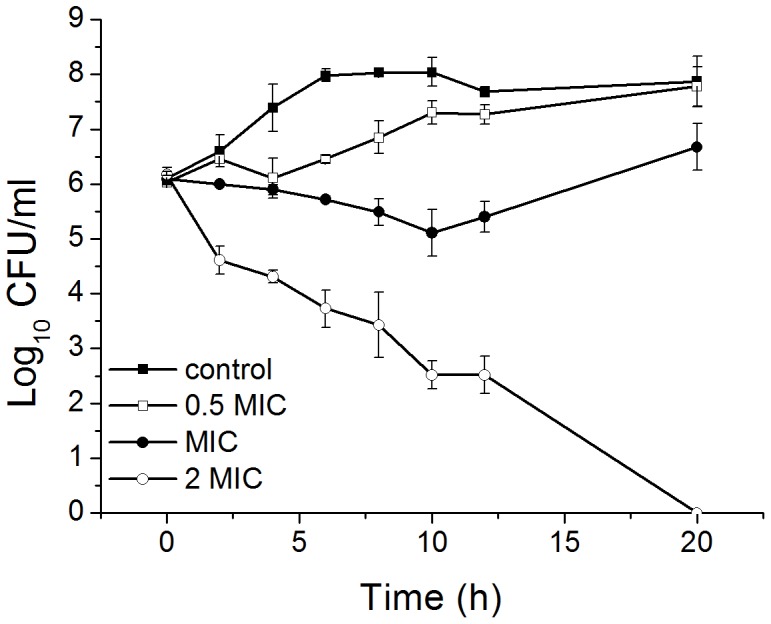
Time-kill curves of trichokonin VI against *B. subtilis*. The concentrations of trichokonin VI used were 0 µM, 12.5 µM, 25 µM and 50 µM, which corresponded to 0, 0.5, 1 and 2 × MIC, respectively.

The time-kill curves of trichokonin VI against *B. subtilis* are shown in Fig. 1. Trichokonin VI exhibited a concentration-dependent antimicrobial activity in the bacterial viability test. Trichokonin VI at a concentration of 0.5 × MIC reduced cell growth in the first few hours when compared with the control; however, the growth curves were similar after 10 h of incubation. Trichokonin VI at the MIC inhibited the growth of *B. subtilis*; however, the bacteria number increased slightly after treatment for 20 h. At a higher concentration (2 × MIC), trichokonin VI led to a progressive decrease in number of bacterial colony forming units, and no living bacteria were detected after 20 h.

### AFM Images of *B. subtilis*

**Figure 2:**
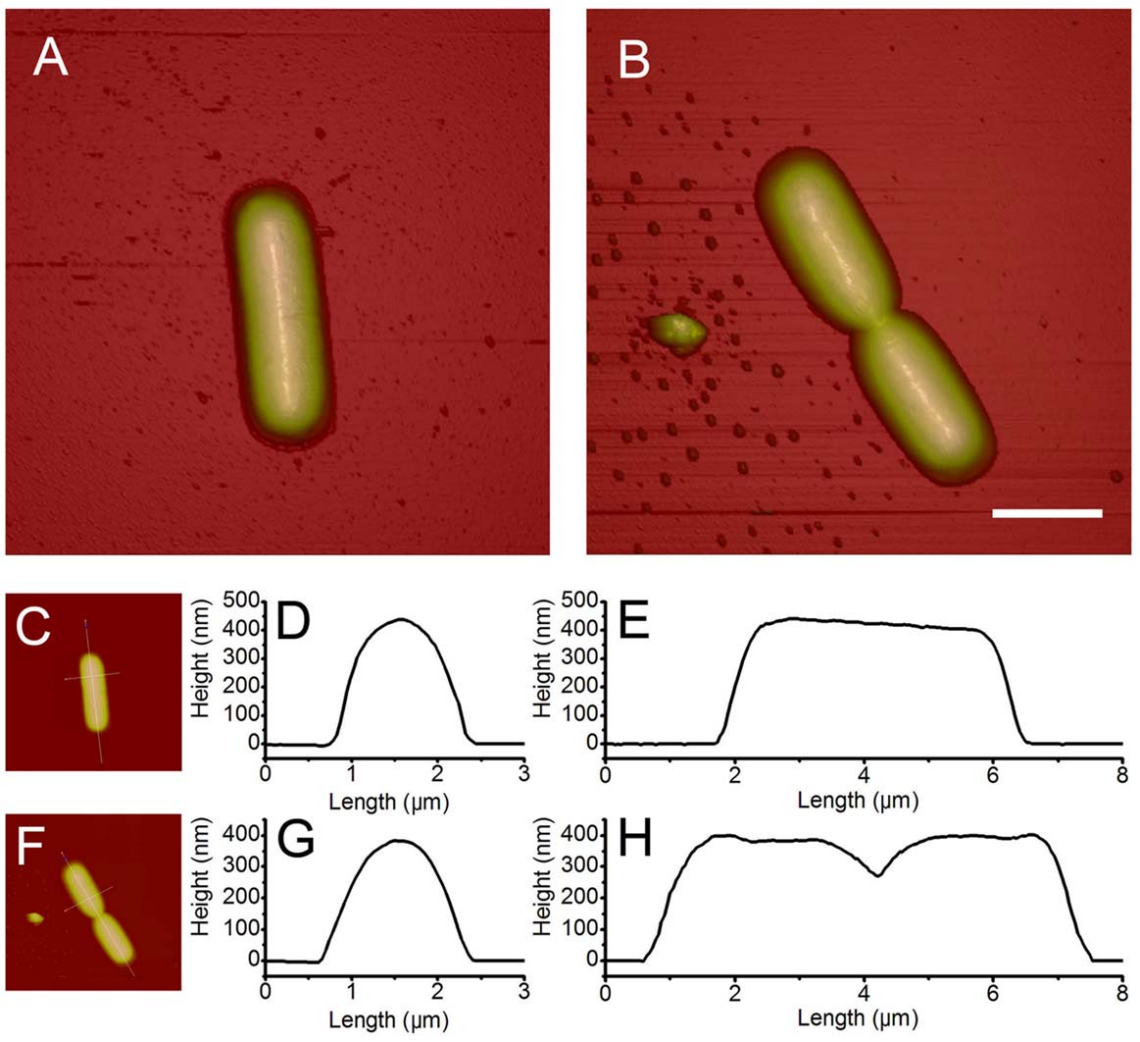
Morphology and section analysis of *B. subtilis*. A and B are 3-dimensional AFM height images of *B. subtilis* cells, and B shows a dividing cell. C and F show the 2-dimensional height data from A and B, respectively, indicating the cross section position. D and E are the cross sections of the image indicated in C; and G and H are the cross sections of the image indicated in F. Scale bar is 2 µm.

Images of the *B. subtilis* cells freshly collected from the bacterial culture were acquired (Fig. 2). The surfaces of cells are reasonably smooth with a bacillary shape. No visible pores or ruptures could be observed in all examined cells. Cross sections of the bacterial cells were acquired. The measured length, width and height, shown in Fig. 2A, were 4.4, 1.3, and 0.44 µm, respectively, which are comparable to the reported dimensions [24]. However, the bacteria were observed to have a variation in length, and this variation may be a result of detecting living bacteria at different growth stages. Trace amount of methanol in trichokonin VI solution had no obvious effect on the morphology of the *B. subtilis* cells (Fig. S1). Suspending bacteria in deionized water would bring about the hypo-osmotic shock to the bacterial cells. However, the sample preparation step in this experiment was short and no obvious random damage to the bacteria cells of *B. subtilis* was observed (Fig. S2).

### AFM Images of *B. subtilis* Cells Treated with Trichokonin VI

**Figure 3:**
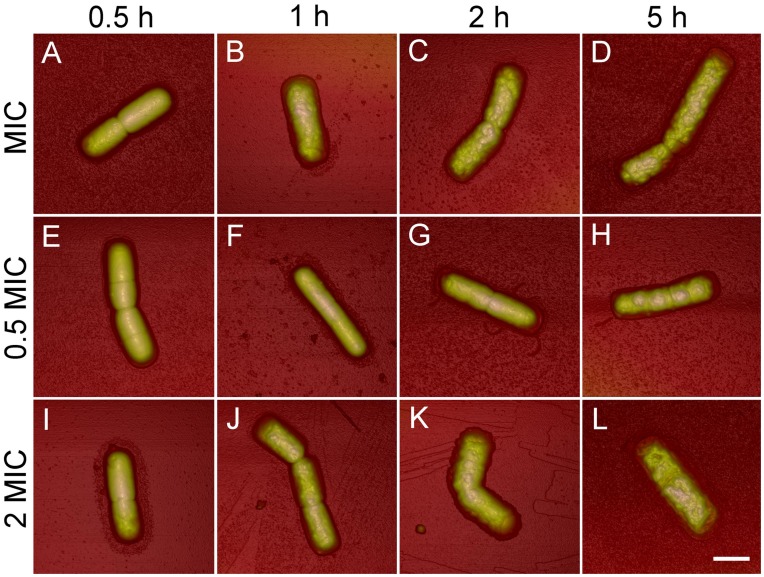
The 3-dimensional height images of *B. subtilis* treated with trichokonin VI. *B. subtilis* were treated with trichokonin VI at the MIC (25 µM, top row), 0.5 × MIC (12.5 µM, middle row) and 2 × MIC (50 µM, bottom row) for 0.5 h (first column), 1 h (second column), 2 h (third column) and 5 h (fourth column). Scale bar is 2 µm.

The antimicrobial effect of trichokonin VI on *B. subtilis* at different treatment times was monitored. For each sample, randomly selected cells were examined and analyzed. *B. subtilis* was treated with trichokonin VI at the MIC for a period of 0.5, 1, 2 and 5 h, and the treated cells were collected and imaged. After treatment, the bacterial cells retained their rod-like form (Fig. 3A–D). However, minor corrugations to the bacterial surface could be distinguished after a 0.5-h incubation. The corrugation was more evident as the incubation time was increased. Treatment for 2 h or longer induced greater disruption in the cell morphology and collapse of the cell wall was observed. After incubation, the height of the *B. subtilis* cells declined from approximately 500 nm to less than 400 nm (Fig. S3), which suggests a dramatic decrease in cell volume after trichokonin VI treatment.

Changes to the surface characteristics of *B. subtilis* as a result of trichokonin VI treatment are obvious. Treatment at 0.5 × MIC resulted in minor surface perturbations after 2 h, and cells with more obvious changes could be detected after 5 h (Fig. 3E–H and Fig. S4). Upon treatment at 2 × MIC, a more pronounced collapse of the cell wall could be detected (Fig. 3I–L), and collapse of the apical end of the cell was evident. Surface characteristics changed after the first 0.5 h of incubation. After 5 h of incubation, the height of the treated bacterial cells declined to less than 300 nm (Fig. S5), suggesting a greater reduction in cell volume. The formation of granules on the *B. subtilis* cells after treatment with trichokonin VI was observed, and the granules are most likely formed from the condensation of cells because of the leakage of cellular materials.

Bacterial cells diluted only with growth broth and incubated for different time periods were set as control. These bacterial cells were also imaged (Fig. S6). The morphologies of the bacteria were comparable to those freshly collected from bacteria culture. No obvious morphological collapse was observed. Thus, the results indicated that the alteration in bacterial morphological properties was induced by trichokonin VI.

### Surface Roughness Analysis

**Figure 4:**
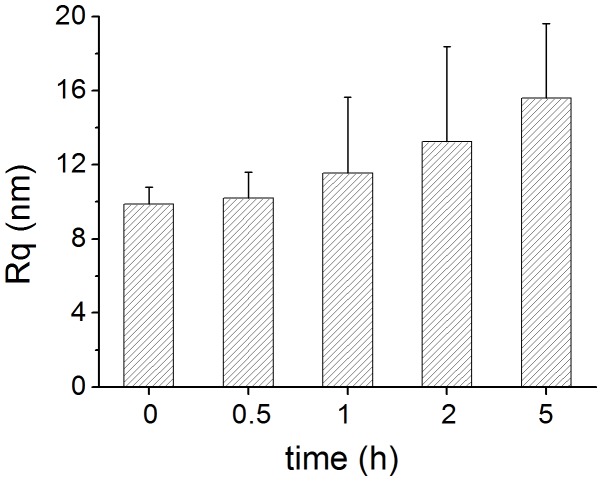
Surface roughness of *B. subtilis* treated with trichokonin VI at the MIC for different periods of time. *Rq* was calculated in fixed-square size with a side length of 400 nm.

*B. subtilis* cells treated with trichokonin VI showed alterations in surface characteristics compared with untreated cells. Images were analyzed by calculating the roughness of the bacterial cell surface. Surface roughness increased as the treatment time increased (Fig. 4). The surface roughness, *Rq*, of the untreated cells over an area of 400 × 400 nm was calculated to be 9.9±0.9 nm. After incubation with trichokonin VI for 0.5 h, *Rq* slightly increased (10.2±1.4 nm) compared to the untreated cells. After 1 h of treatment, *Rq* increased to11.5±4.1 nm and it increased to 15.6±4.0 nm after 5 h of incubation. The results showed that the antimicrobial effects of trichokonin VI on *B. subtilis* are time dependent.

### Mechanical Properties of the *B. subtilis* Cells

**Figure 5:**
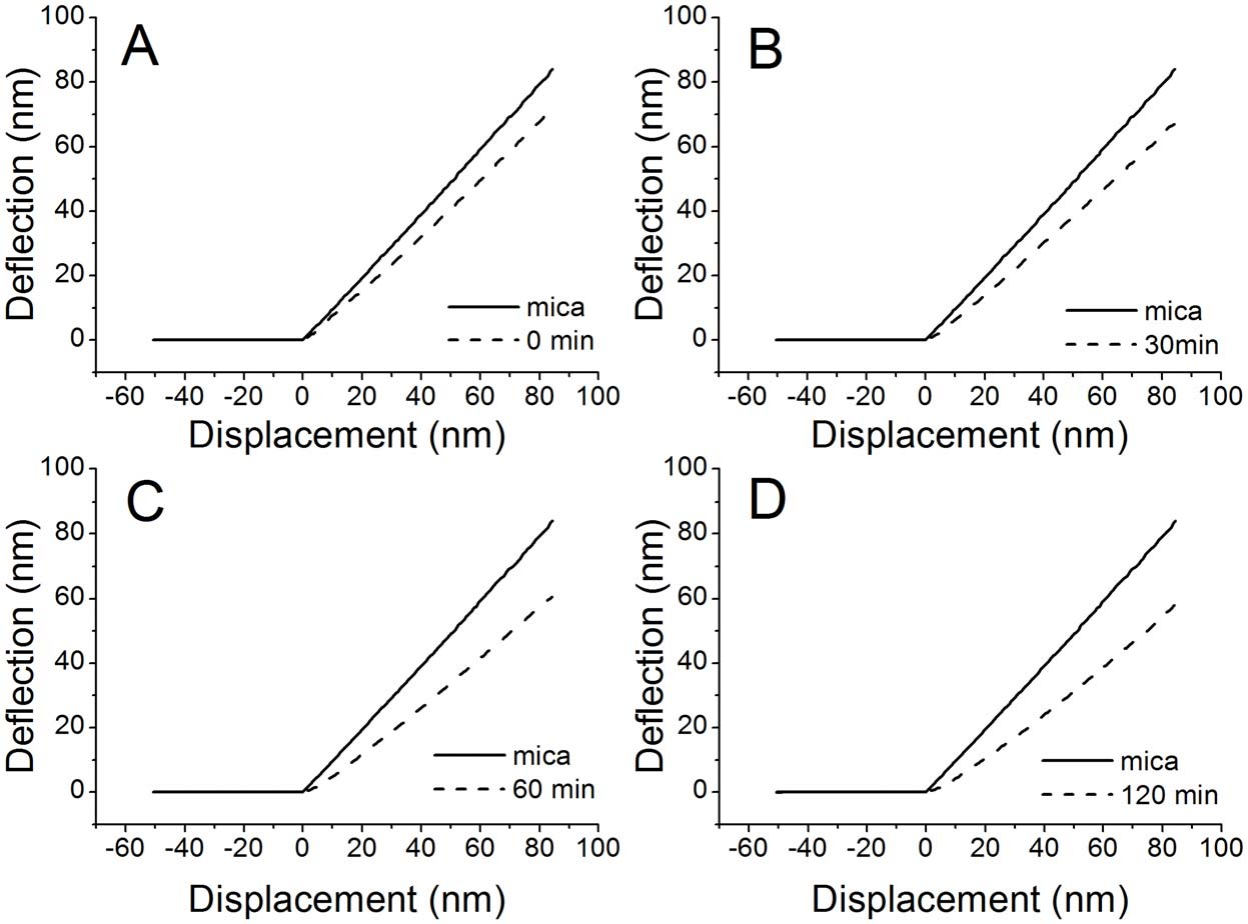
Representative approaching branch of force-distance curves on mica and *B. subtilis*. Curves were collected on mica and *B. subtilis* incubated with trichokonin VI at the MIC (25 µM) for 0 min (A), 30 min (B), 60 min (C), and 120 min (D).

For force-distance measurements, the force curves were collected near the center of the bacterial cells. The force measurements were performed on the *B. subtilis* cells treated with trichokonin VI at the MIC for different incubation times. The spring constants of the *B. subtilis* cells were first calculated. For the untreated cells, the spring constant was 12.1±4.1 N/m (Table 1). The measured value was larger than the values obtained from bacteria in liquid [27]. The influence of trichokonin VI to the nanomechanical properties of *B. subtilis* incubated with trichokonin VI for 0.5, 1 and 2 h was analyzed. It was observed from the force-distance curves that the slopes of the curves on the bacteria are all less steep than on the slopes on mica, and an increased incubation time led to shallower slopes (Fig. 5). Statistical analysis revealed that the spring constant of the cells decreased with an increase in the incubation time. The spring constants of the *B. subtilis* cells treated with trichokonin VI are summarized in Table 1. After a 0.5-h treatment, the spring constant decreased to 9.8±3.8 N/m and further dropped to 7.3±4.1 N/m after a 1-h incubation. When treated with trichokonin VI for 2 h, the spring constant of the *B. subtilis* cells decreased to 6.2±3.1 N/m, or approximately half the spring constant of untreated cells.

**Table 1:** Nanomechanical properties of *B. subtilis* incubated with trichokonin VI at MIC for different time periods.

	Control	30 min	60 min	120 min
**spring constant (N/m)**	12.1±4.1	9.8±3.8	7.3±4.1	6.2±3.1
**adhesive force (nN)**	26.4±3.3	23.2±4.4	25.9±8.5	44.5±11.9
**adhesive energy (×10^−18^ J)**	406±61	339±127	427±183	753±332

The retract branch of the force curves between the AFM tip and *B. subtilis* cells treated with trichokonin VI for different time periods showed that the adhesion force had only minor changes compared with the untreated cells in the first 1 h. However, the force dramatically increased after 2 h of incubation with trichokonin VI (Fig. S7 and Table 1), which suggests marked changes to the bacterial surface properties. Work performed by adhesion forces, such as adhesion energy, was also calculated (Table 1), and these forces exhibited similar trends as the adhesion force upon trichokonin VI treatment.

### Leakage of Cellular UV-absorbing Materials

**Figure 6:**
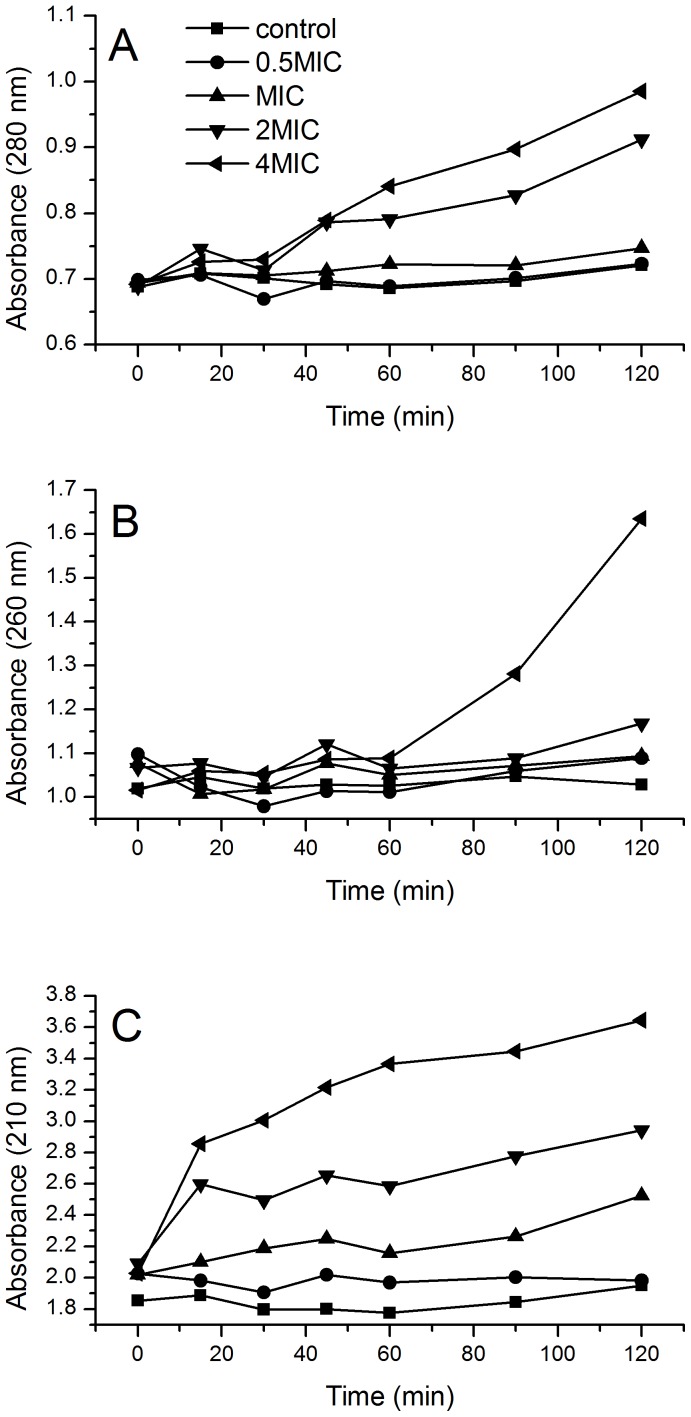
Release of UV-absorbing substances from *B. subtilis* treated with trichokonin VI at different concentrations. *B. subtilis* were treated with trichokonin VI at 0.5 × MIC (12.5 µM), MIC (25 µM), 2 × MIC (50 µM) and 4 × MIC (100 µM). Absorbance was measured at 280 nm (A), 260 nm (B) and 210 nm (C).

Leakage of cellular UV-absorbing substances is an indicator of changes in membrane permeability. Thus, we monitored the UV-absorption of the supernatant after *B. subtilis* cells were treated with trichokonin VI. Low doses of trichokonin VI had little effect on the leakage of cellular UV-absorbing substances (Fig. 6). An increase in absorption at 260 and 280 nm could only be observed when *B. subtilis* cells were treated with high concentrations of trichokonin VI for a relatively long time. However, the absorption at 210 nm increased after incubation for only 20 min at high trichokonin VI concentrations. For treatment with low trichokonin VI concentrations, variations in the 210-nm absorption values after long incubation times were observed.

### Membrane Permeabilization Test

**Figure 7:**
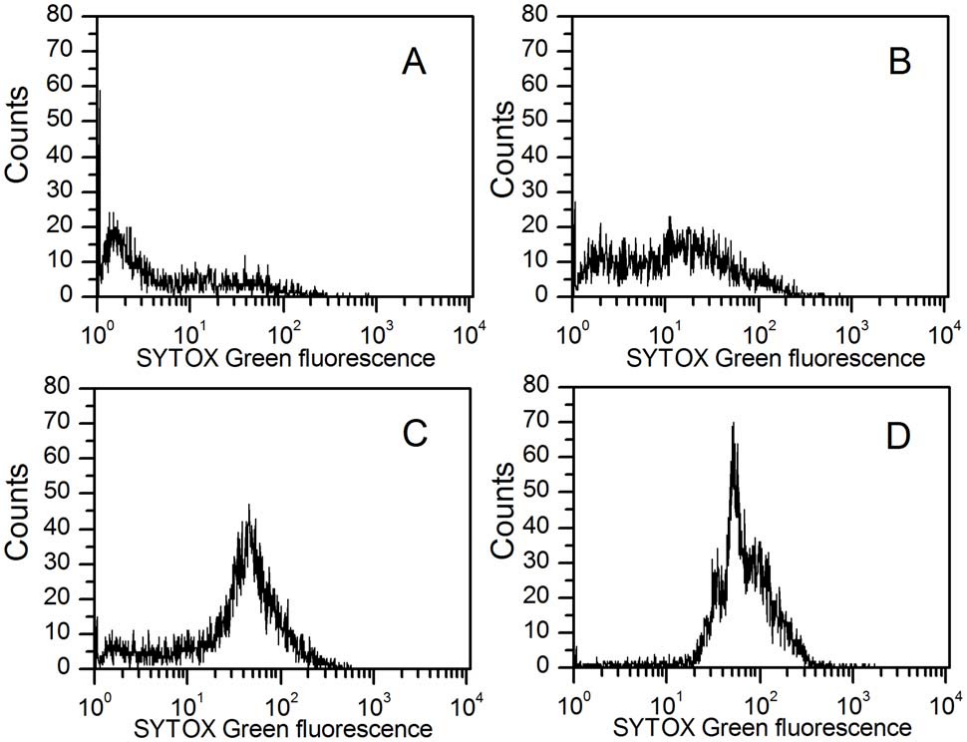
Membrane permeabilization effects of trichokonin VI on *B. subtilis* cells. The *B. subtilis* cells were stained with SYTOX Green and analyzed with flow cytometry. The bacteria were untreated (A), treated with trichokonin VI at a concentration of 0.5 × MIC (B), MIC (C) or 2 × MIC (D) for 1 h.

SYTOX Green is a high affinity nucleic acid dye that becomes more fluorescent when bound to DNA [32], [33]. SYTOX Green does not cross the membranes of live cells but can enter into cells with a compromised membrane [32], [33]. When SYTOX Green was applied to the healthy bacterial cells which were untreated with trichokonin VI, a weak but detectable fluorescence emission could be noticed (Fig. 7A). This weak fluorescence resulted from the binding of dye to bacterial surfaces, which is consistent with previous report [34]. Fluorescence intensity from *B. subtilis* cells treated with trichokonin VI at low concentrations appeared similar to the untreated cells. However, when the bacterial cells were treated with trichokonin VI at MIC or higher, an enhancement of SYTOX Green fluorescence could be observed (Fig. 7B), suggesting that the membranes of *B. subtilis* cells have been disrupted by the membrane-active compound trichokonin VI at these concentrations.

## Discussion

Trichokonin VI was determined to be effective against Gram-positive *B. subtilis* with a MIC of 25 µM. The MIC of trichokonin VI against *B. subtilis* was comparable to some AMPs, such as Magainin2 [29], and higher than other AMPs, such as LL-37 [29] and MSI-594 [31]. Here, we combined AFM and other methods to investigate the antimicrobial effects of trichokonin VI on *B. subtilis* and determined that the peptaibol induced changes to the morphological and mechanical properties of the bacterium as well as permeabilized the bacterial membrane. Our results suggest a concentration- and time-dependent mode of action in modifying the morphological and nanomechanical properties of *B. subtilis* cells.

Microscopic methods are important tools for studying the interactions between AMPs and microorganisms. AFM is among the most powerful of the microscopic tools. Bacterial ultrastructures can be investigated with a high resolution, and their mechanical properties can be monitored by AFM. Height and surface roughness of the cells can be analyzed from the AFM images. Cellular morphological and mechanical properties are directly related to the mode of action of the antimicrobial agents; therefore, these advantages, in combination with other tools, may help us better understand the antimicrobial mechanisms of AMPs. Efforts have been made to evaluate the morphological changes of the bacteria treated with AMPs. Most of this work has focused on Gram-negative bacteria, which has an outer membrane that surrounds the peptidoglycan layer. However, there has been little work on the application of AFM to monitor the antimicrobial effects of AMPs on Gram-positive bacteria.

Imaging with a 3-dimensional scale using AFM allows for analysis of the surface roughness of bacterial cells. Roughness analysis of the cell surface can help us to understand and quantify the cellular alteration process, especially the detection of subtle changes caused by AMPs after treatment. It is widely accepted that AMPs interact with membranes, and the cell membrane of Gram-positive bacteria is surrounded by a thick layer of peptidoglycan. Therefore, changes in the roughness properties might be due to changes beneath the peptidoglycan layer. Along with the increased treatment time, surface roughness of *B. subtilis* cells gradually increased. Alteration to the surface roughness was a time-dependent process, which is in agreement with the morphological observations.

The results show that the mechanical strength of *B. subtilis* was altered under treatment with trichokonin VI. The mechanical properties of bacteria usually originate from the cell wall and turgor pressure of the cell [35]. Maintaining the bacterial structure necessarily involves the mechanical strength of the cell, which needs the cell wall to constrain the cellular contents under turgor pressure and defines the cell shape. Turgor pressure is generated from the cellular contents in the cytoplasm, which push against the cytoplasmic membrane and peptidoglycan wall. The permeabilization experiments indicated that the membranes are the target of trichokonin VI. With an increase in incubation time, it was observed that the spring constant of the *B. subtilis* cells gradually decreased. Thus, alterations in the mechanical properties are most likely due to the leakage of the intracellular materials, which led to changes in the turgor pressure, and resulted in a reduction in cellular elasticity as well as a collapse of the cell structure. From the retract part of the force-distance curve, adhesion forces between the AFM tip and bacterial surface can be measured. The adhesion force may originate from the interactions between the probe tip and molecules on the bacterial cell wall, including peptidoglycans, leaked biomacromolecules and water layers. A change in the adhesion force is an indicator of a change in the cell surface characteristics.

Research on morphological alterations has clearly exhibited a time- and concentration-dependent process for the antimicrobial effects of trichokonin VI on *B. subtilis*. Structural collapse usually results from leakage of intracellular materials or starvation. Structural shriveling of the bacterial cells could be observed in nutrient-free buffer [13]. In our experiments, the collapse was not a result of starvation (Fig. S6). Alves et al. discovered that the AMPs induced a collapse in the septional region of the *E. coli* envelopes [13]. However, in the case of *B. subtilis*, it is interesting that the polar region of the *B. subtilis* cells appears to be sensitive to attack and is dependent of the trichokonin VI concentrations used in the experiments.

At 0.5 × MIC, the effects of trichokonin VI on *B. subtilis* cells are less clear. Minor morphological alterations could be observed after 2 h of treatment in the AFM images. After longer treatment times, the cell surface became rougher and section analysis showed a minor reduction in height, which indicated a loss of cellular volume. The minor morphological alterations in the *B. subtilis* cells suggest that trichokonin VI only has a slight effect on the bacteria at this concentration. The growth of *B. subtilis* could not be completely inhibited, which was determined using the time-killing assay.

At higher trichokonin VI concentrations (MIC), the antimicrobial effects on *B. subtilis* cells are much more obvious in the AFM experiments. After the first 0.5 h, only a slight collapse could be detected. After 1 h of treatment, granules were detected. A reduction in cell volume suggests that leakage of the intracellular material was occurring, and the changes in the mechanical properties suggest a decrease in bacterial turgor pressure, which was also a potential result from the leakage of the intracellular materials. Small molecules were allowed to cross the cell membrane, which indicates that the membranes have been permeabilized. Permeabilization of the membrane led to a reduction in cell volume, which caused alterations in morphology, surface roughness, and resulted in cell death.

When the concentration of trichokonin VI was increased to 2 × MIC or higher, more profound changes were detected. The AFM images show that the collapse of the cell structure was more obvious and led to the appearance of more granules on cellular surfaces. Considering the profound reduction in cell height, which was determined using section analysis, the granules are most likely are result of the collapse of the cell structure. Detection of leakage of cellular materials showed that trichokonin VI severely permeabilized the membrane at high concentrations, which caused a much quicker reduction in cellular volume and more profound changes in cell morphology.

In our leakage and membrane permeabilization experiments, the results indicated that the membrane did not allow small organic molecules to cross at low concentrations. At higher concentrations, the membranes were permeabilized and small molecules were allowed to cross the membrane. Trichokonin VI has a helical structure, which is a requirement for channel forming peptides, and the helical structure of trichokonin VI was determined using circular dichroism [36]. Furthermore, trichokonin VI has a length of 20 amino acid residues. Works on other helical peptaibols indicated that the long chain is also a requirement for the peptaibols to be able to penetrate the lipid bilayers and form channels [37], [38].Thus, based on our results and previous studies, it is possible that the target of trichokonin VI is the membrane of bacterial cells, and leakage of intracellular materials induced by trichokonin VI appears to be the reason for the changes in the morphological and nanomechanical properties probed by AFM.

In summary, we have demonstrated that trichokonin VI, a peptaibol isolated from *T. pseudokoningii*, is effective against the Gram-positive bacterium *B. subtilis*. Morphological and mechanical studies observed a concentration- and time-dependent effect against the *B. subtilis* cells. Nanoindentation experiments revealed a progressive decrease in the stiffness of the cells. Furthermore, we monitored the membrane permeabilization effect and suggest that leakage of intracellular materials is a potential mechanism of action of trichokonin VI on *B. subtilis*.

## Supporting Information

Figure S1 **Effect of methanol on** ***B. subtilis*** **cells.** When preparing trichokonin VI stock solutions, 10 mg of trichokonin VI was dissolved in methanol (0.1 ml), and the solution was diluted with Milli-Q water (4.9 ml) to a concentration of 2 mg/ml as a stock solution. When trichokonin VI stock solutions were diluted to the MIC, the solution contained methanol at a concentration of 0.05% (v/v). Thus, it is necessary to examine whether trace amounts of methanol had any effect on *B. subtilis*. We incubated *B. subtilis* cells (10^6^ CFU/ml) with methanol at a concentration of 0.05% (A) and 0.2% (B) (v/v), and these concentrations correspond to methanol content at the MIC and 4 × MIC. Samples were collected after 5 h of treatment. Cells were centrifuged at 7,000 g for 10 min and suspended in Milli-Q water. The cells were imaged by AFM, and the representative results are shown. As shown in the figures, *B. subtilis* cells retained their smooth surfaces and rod shape, and the sizes of the treated cells are comparable to that of the untreated cells (Fig. 2), which suggests that methanol at the concentrations used in our experiments had no visible influence on the *B. subtilis* cells.
(TIF)

Figure S2 **Effect of water on** ***B. subtilis*** **cells during sample preparation.** Suspending the bacteria in deionized water would bring about the hypo-osmotic shock to the bacteria cells. To check the osmotic effect of incubation with water on the morphology of *B. subtilis*, we incubated *B. subtilis* in Milli-Q water for 0.5 h (A) and 1 h (B). These bacterial cells appeared intact with no visible holes, granules, or breakages in the cell envelop, and the morphologies of the bacteria are distinctly different from those treated by antimicrobial peptide. Thus we consider that this sample preparation step did not leave to random damage to the bacteria cells.
(TIF)

Figure S3 **Section analysis of** ***B. subtilis*** **treated with trichokonin VI at the MIC for different incubation times.** *B. subtilis* were treated with trichokonin VI at the MIC (25 µM) for 0.5 h (A, B), 1 h (C, D), 2 h (E, F) and 5 h (G, H). A and B are cross sections of the image in Fig. 3A; C and D are cross sections of the image in Fig. 3B; E and F are cross sections of the image in Fig. 3C; and G and H are cross sections of the image in Fig. 3D. A, C, E and G are section profiles along the short axis of the bacterial cells. B, D, F and H are section profiles along the long axis of the bacterial cells.
(TIF)

Figure S4 **Section analysis of** ***B. subtilis*** **treated with trichokonin VI at 0.5 × MIC for different times.** *B. subtilis* were treated with trichokonin VI at 0.5 × MIC (12.5 µM) for 0.5 h (A, B), 1 h (C, D), 2 h (E, F) and 5 h (G, H). A and B are cross sections of the image in Fig. 3E; C and D are cross sections of the image in Fig. 3F; E and F are cross sections of the image in Fig. 3G; G and H are cross sections of the image in Fig. 3H. A, C, E and G are section profiles along the short axis of the bacterial cells. B, D, F and H are section profiles along the long axis of the bacterial cells.
(TIF)

Figure S5 **Section analysis of** ***B. subtilis*** **treated with trichokonin VI at 2 × MIC for different times.** *B. subtilis* were treated with trichokonin VI at 2 × MIC (50 µM) for 0.5 h (A, B), 1 h (C, D), 2 h (E, F) and 5 h (G, H). A and B are cross sections of the image in Fig. 3I; C and D are cross sections of the image in Fig. 3J; E and F are cross sections of the image in Fig. 3K; and G and H are cross sections of the image in Fig. 3L. A, C, E and G are section profiles along the short axis of the bacterial cells. B, D, F and H are section profiles along the long axis of the bacterial cells.
(TIF)

Figure S6 **Representative images of the control** ***B. subtilis*** **cells which were not treated with trichokonin VI.** The *B. subtilis* cells which were not treated with trichokonin VI were set as control samples to those which were treated with trichokonin VI. The morphologies of the control bacterial cells incubated for different time periods were monitored. The morphological properties of the bacterial cells incubated for 2 h (A) or 5 h (B) was comparable to that freshly collected from broth medium (Fig. 2). Scale bar, 2 µm.
(TIF)

Figure S7 **Representative retract branches of force-distance curves on mica and** ***B. subtilis.*** Curves were collected on mica and *B. subtilis* incubated with trichokonin VI at the MIC (25 µM) for 0 h (A), 0.5 h (B), 1 h (C) and 2 h (D). The jump-off contact point represents the adhesion force between the probe tip and bacterial surface.
(TIF)
