# Supplementary figures and images for: Antimicrobial Peptide Trichokonin VI-Induced Alterations in the Morphological and Nanomechanical Properties of Bacillus subtilis
Source: PLoS One. 2012 Sep 25;7(9):e45818. doi: 10.1371/journal.pone.0045818 (PMC3458079; doi:10.1371/journal.pone.0045818)

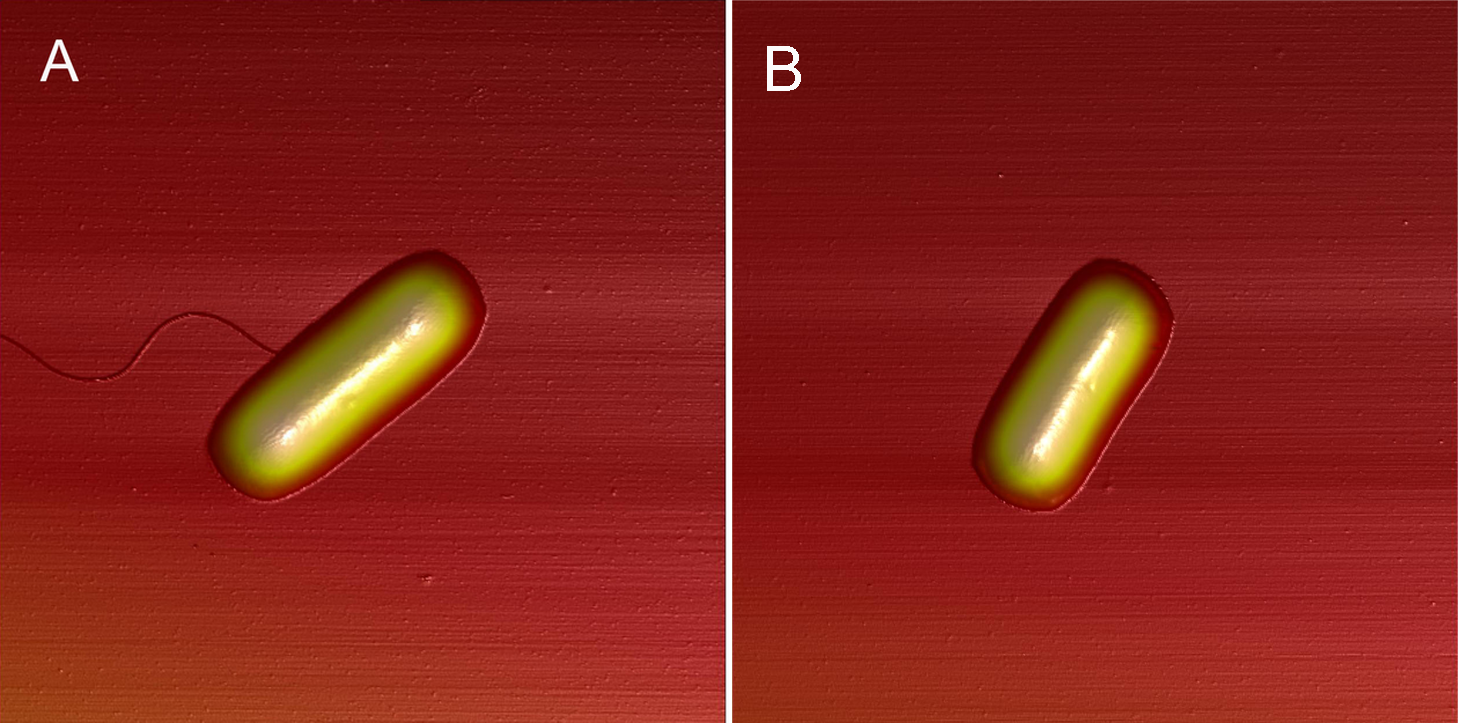

Supplement: Figure S1 — Effect of methanol on B. subtilis cells. When preparing trichokonin VI stock solutions, 10 mg of trichokonin VI was dissolved in methanol (0.1 ml), and the solution was diluted with Milli-Q water (4.9 ml) to a concentration of 2 mg/ml as a stock solution. When trichokonin VI stock solutions were diluted to the MIC, the solution contained methanol at a concentration of 0.05% (v/v). Thus, it is necessary to examine whether trace amounts of methanol had any effect on B. subtilis. We incubated B. subtilis cells (106 CFU/ml) with methanol at a concentration of 0.05% (A) and 0.2% (B) (v/v), and these concentrations correspond to methanol content at the MIC and 4 × MIC. Samples were collected after 5 h of treatment. Cells were centrifuged at 7,000 g for 10 min and suspended in Milli-Q water. The cells were imaged by AFM, and the representative results are shown. As shown in the figures, B. subtilis cells retained their smooth surfaces and rod shape, and the sizes of the treated cells are comparable to that of the untreated cells (Fig. 2), which suggests that methanol at the concentrations used in our experiments had no visible influence on the B. subtilis cells. (TIF) [file pone.0045818.s001.tif]

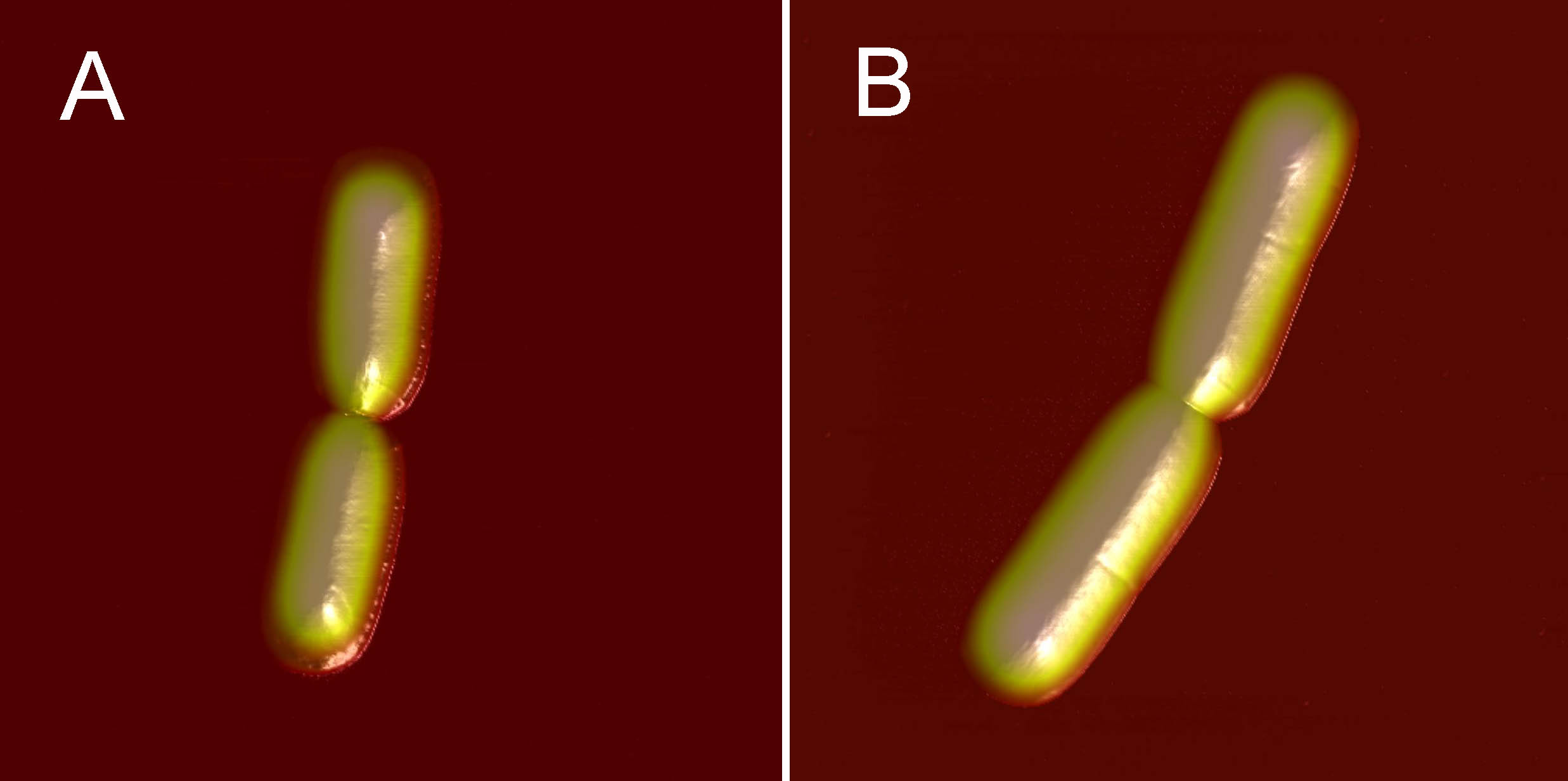

Supplement: Figure S2 — Effect of water on B. subtilis cells during sample preparation. Suspending the bacteria in deionized water would bring about the hypo-osmotic shock to the bacteria cells. To check the osmotic effect of incubation with water on the morphology of B. subtilis, we incubated B. subtilis in Milli-Q water for 0.5 h (A) and 1 h (B). These bacterial cells appeared intact with no visible holes, granules, or breakages in the cell envelop, and the morphologies of the bacteria are distinctly different from those treated by antimicrobial peptide. Thus we consider that this sample preparation step did not leave to random damage to the bacteria cells. (TIF) [file pone.0045818.s002.tif]

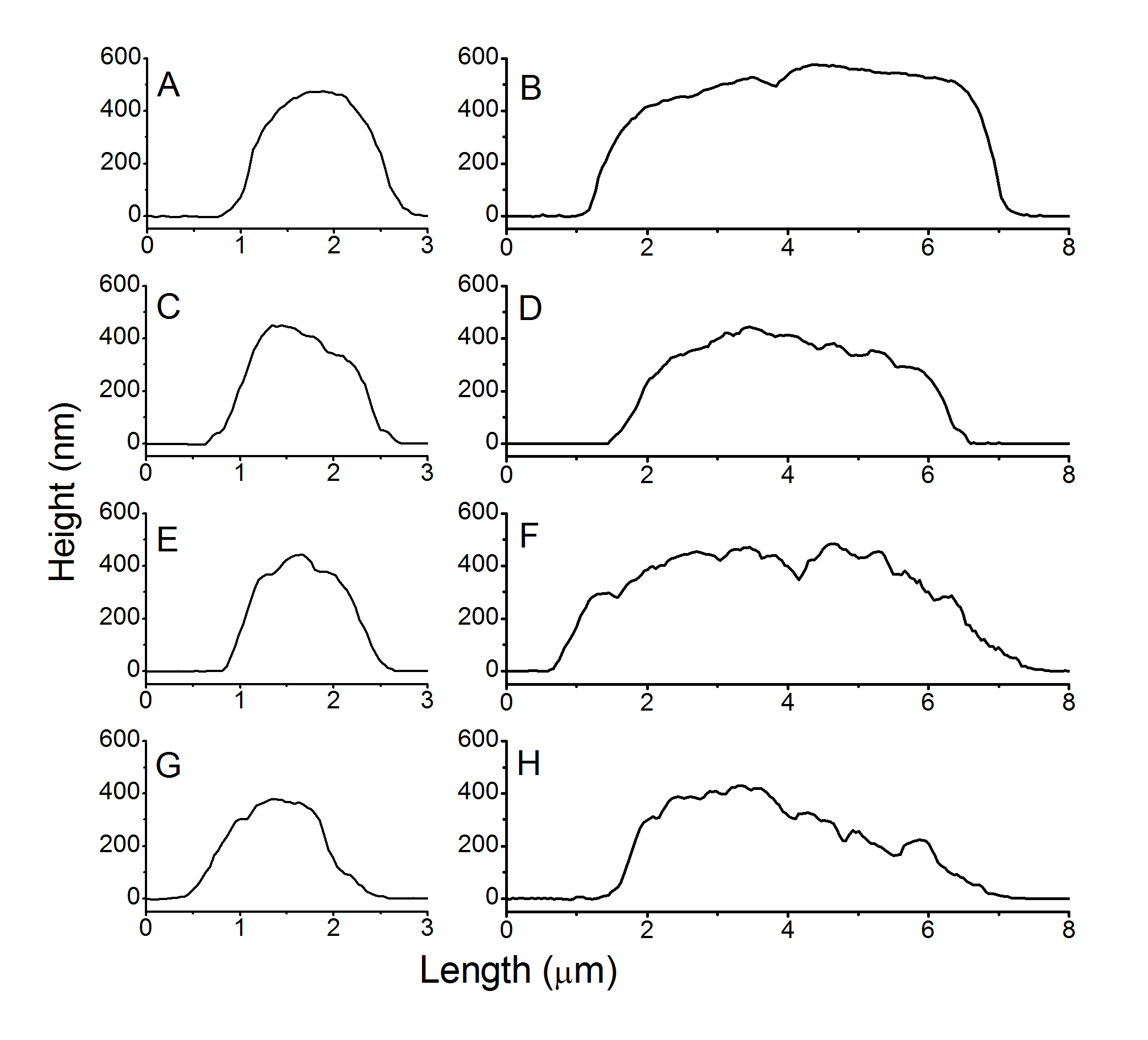

Supplement: Figure S3 — Section analysis of B. subtilis treated with trichokonin VI at the MIC for different incubation times. B. subtilis were treated with trichokonin VI at the MIC (25 µM) for 0.5 h (A, B), 1 h (C, D), 2 h (E, F) and 5 h (G, H). A and B are cross sections of the image in Fig. 3A; C and D are cross sections of the image in Fig. 3B; E and F are cross sections of the image in Fig. 3C; and G and H are cross sections of the image in Fig. 3D. A, C, E and G are section profiles along the short axis of the bacterial cells. B, D, F and H are section profiles along the long axis of the bacterial cells. (TIF) [file pone.0045818.s003.tif]

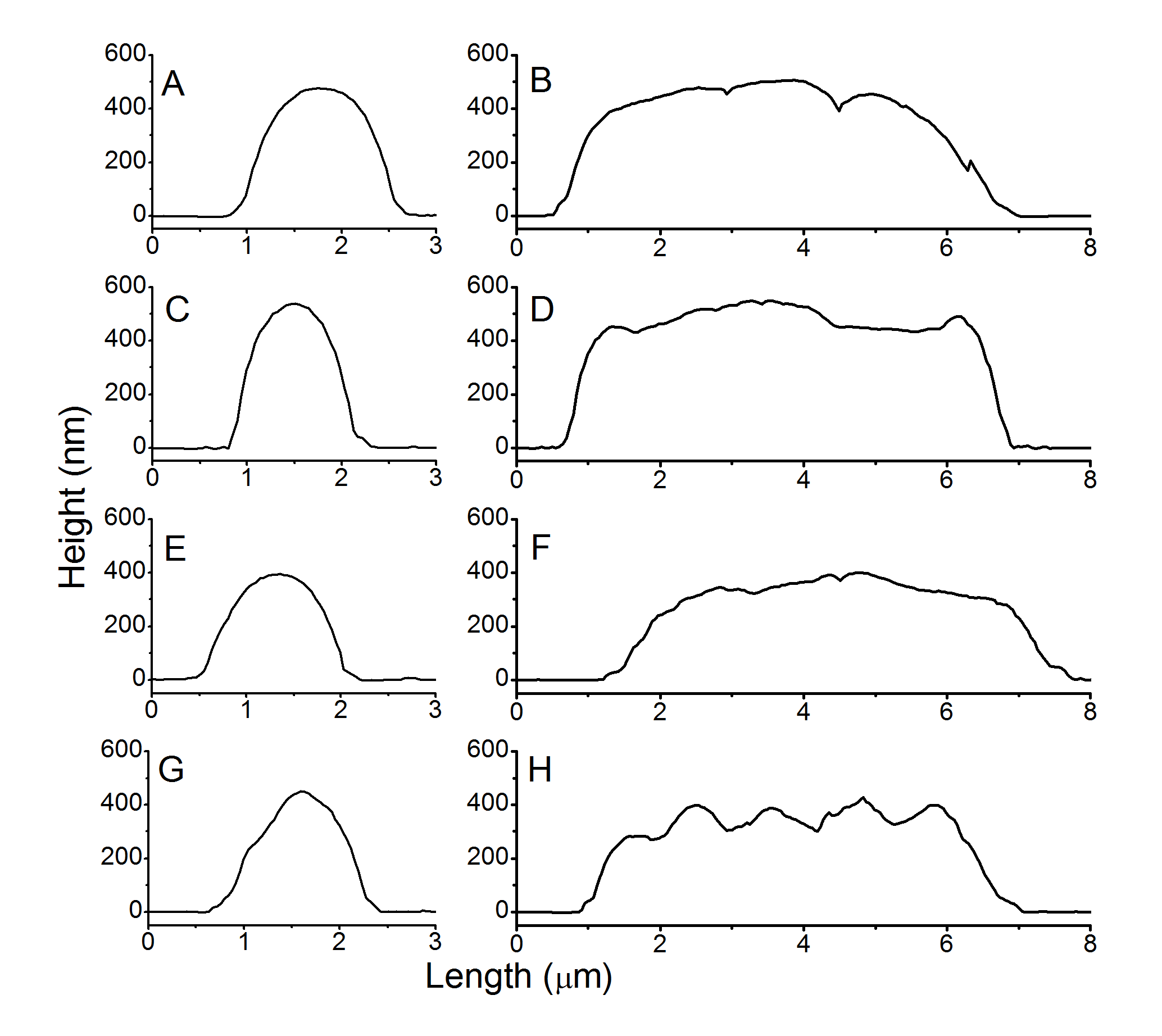

Supplement: Figure S4 — Section analysis of B. subtilis treated with trichokonin VI at 0.5 × MIC for different times. B. subtilis were treated with trichokonin VI at 0.5 × MIC (12.5 µM) for 0.5 h (A, B), 1 h (C, D), 2 h (E, F) and 5 h (G, H). A and B are cross sections of the image in Fig. 3E; C and D are cross sections of the image in Fig. 3F; E and F are cross sections of the image in Fig. 3G; G and H are cross sections of the image in Fig. 3H. A, C, E and G are section profiles along the short axis of the bacterial cells. B, D, F and H are section profiles along the long axis of the bacterial cells. (TIF) [file pone.0045818.s004.tif]

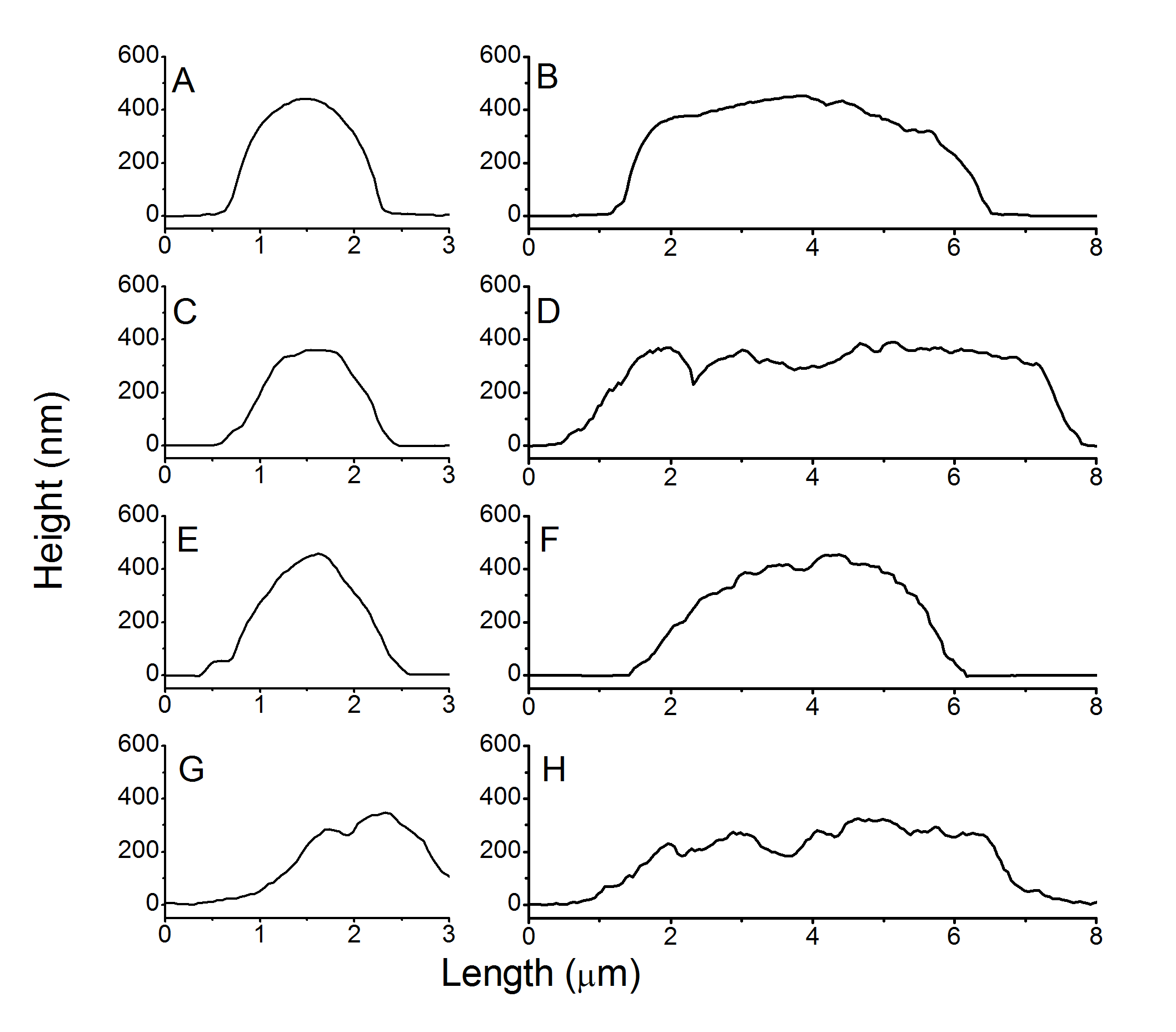

Supplement: Figure S5 — Section analysis of B. subtilis treated with trichokonin VI at 2 × MIC for different times. B. subtilis were treated with trichokonin VI at 2 × MIC (50 µM) for 0.5 h (A, B), 1 h (C, D), 2 h (E, F) and 5 h (G, H). A and B are cross sections of the image in Fig. 3I; C and D are cross sections of the image in Fig. 3J; E and F are cross sections of the image in Fig. 3K; and G and H are cross sections of the image in Fig. 3L. A, C, E and G are section profiles along the short axis of the bacterial cells. B, D, F and H are section profiles along the long axis of the bacterial cells. (TIF) [file pone.0045818.s005.tif]

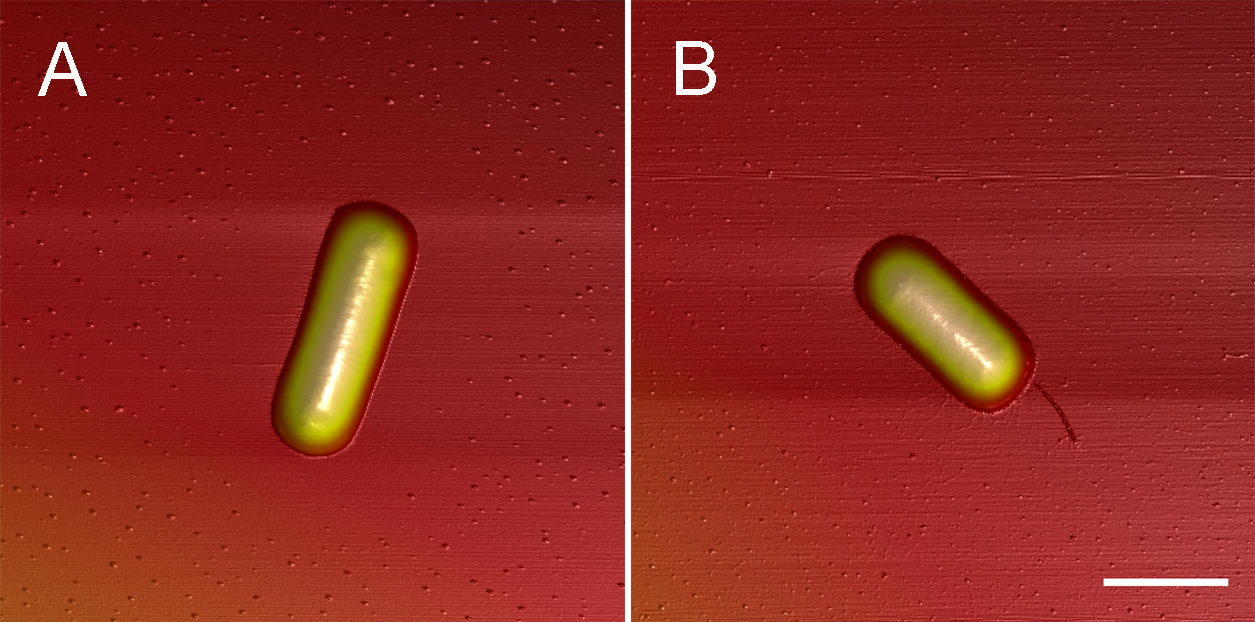

Supplement: Figure S6 — Representative images of the control B. subtilis cells which were not treated with trichokonin VI. The B. subtilis cells which were not treated with trichokonin VI were set as control samples to those which were treated with trichokonin VI. The morphologies of the control bacterial cells incubated for different time periods were monitored. The morphological properties of the bacterial cells incubated for 2 h (A) or 5 h (B) was comparable to that freshly collected from broth medium (Fig. 2). Scale bar, 2 µm. (TIF) [file pone.0045818.s006.tif]

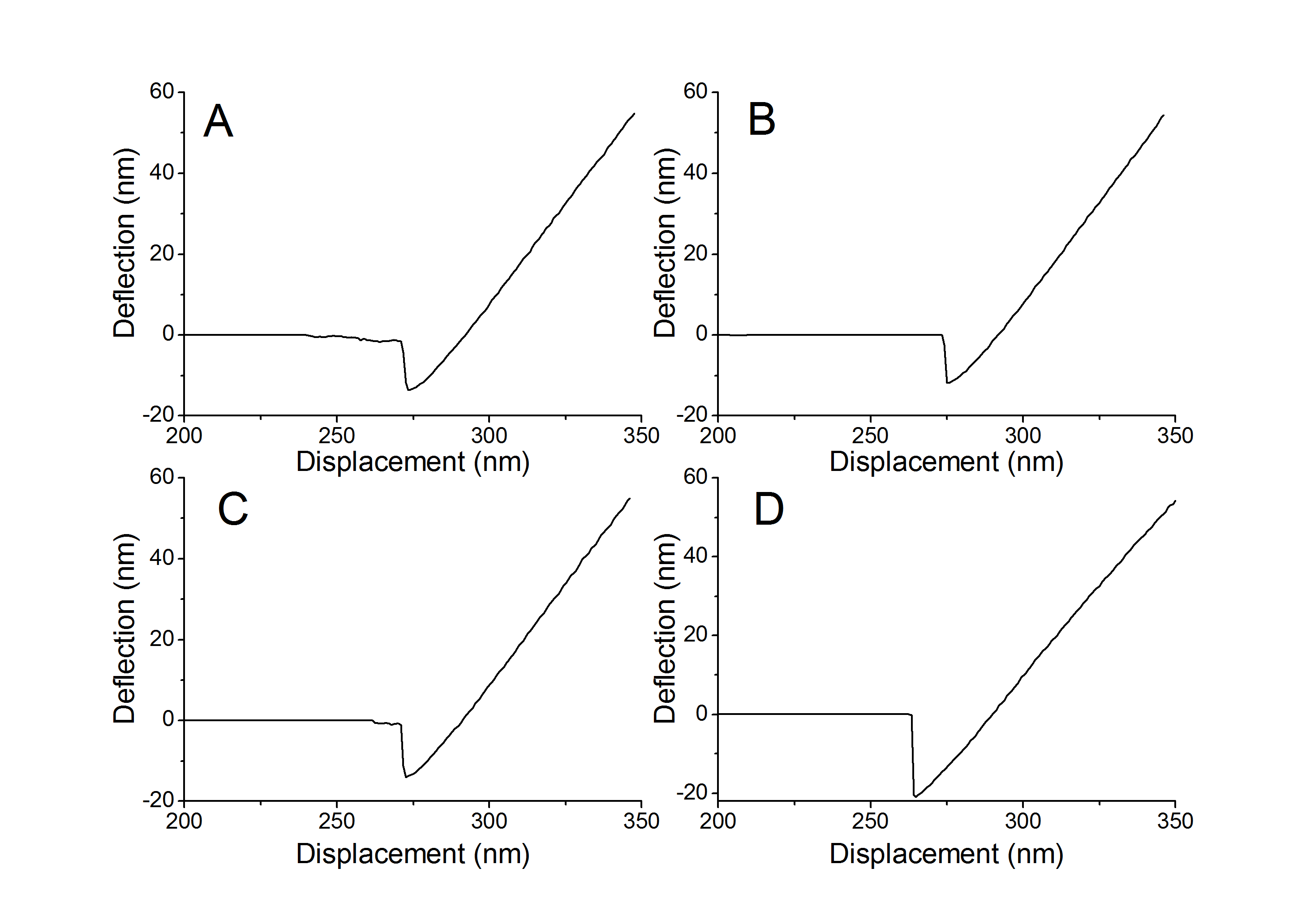

Supplement: Figure S7 — Representative retract branches of force-distance curves on mica and B. subtilis. Curves were collected on mica and B. subtilis incubated with trichokonin VI at the MIC (25 µM) for 0 h (A), 0.5 h (B), 1 h (C) and 2 h (D). The jump-off contact point represents the adhesion force between the probe tip and bacterial surface. (TIF) [file pone.0045818.s007.tif]
